# Minor histological components predict the recurrence of patients with resected stage I acinar- or papillary-predominant lung adenocarcinoma

**DOI:** 10.3389/fonc.2022.1090544

**Published:** 2022-12-23

**Authors:** Wei Liu, Qian Zhang, Tiantian Zhang, Li Li, Chunhua Xu

**Affiliations:** ^1^ Department of Respiratory Medicine, Affiliated Nanjing Brain Hospital, Nanjing Medical University, Nanjing, Jiangsu, China; ^2^ Clinical Center of Nanjing Respiratory Diseases and Imaging, Nanjing chest hospital, Jiangsu, China

**Keywords:** lung adenocarcinoma, prognosis, stage I, non-predominant patterns, histological subtype

## Abstract

**Objective:**

Invasive lung adenocarcinoma is composed of five different histological subgroups with diverse biological behavior and heterogeneous morphology, the acinar/papillary-predominant lung adenocarcinomas are the most common subgroups and recognized as an intermediate-grade group. In the real world, clinicians primarily consider predominant patterns and ignore the impact of minor components in the prognosis of lung adenocarcinoma. The study evaluated the clinicopathologic characteristics of the lepidic, solid, and micropapillary patterns as non-predominant components and whether the minimal patterns had prognostic value on acinar/papillary-predominant lung adenocarcinomas.

**Methods:**

A total of 153 acinar/papillary-predominant lung adenocarcinoma patients with tumor size ≤4 cm were classified into four risk subgroups based on the presence of lepidic and micropapillary/solid components: MP/S^−^Lep^+^, MP/S^+^Lep^+^, MP/S^−^Lep^−^, and MP/S^+^Lep^−^ groups. The Cox-proportional hazard regression model was used to assess disease-free survival (DFS).

**Results:**

The risk subgroups based on the non-predominant patterns were associated with differentiation (*P* = 0.001), lymphovascular invasion (*P* = 0.001), and recurrence (*P* = 0.003). In univariate analysis, DFS was correlated with non-predominant components (*P* = 0.014), lymphovascular invasion (*P* = 0.001), carcinoembryonic antigen (CEA) (*P* = 0.001), and platelet-to-lymphocyte ratio (PLR) (*P* = 0.012). In the multivariate analysis, non-predominant components (*P* = 0.043) and PLR (*P* = 0.032) were independent prognostic factors for DFS. The 5-year survival rates of MP/S^−^Lep^+^, MP/S^+^Lep^+^, MP/S^−^Lep^−^ and MP/S^+^Lep^−^ subgroups were 93.1%,92.9%,73.1%,61.9%, respectively. The MP/S^−^Lep^+^ subgroup had the favorable prognosis than MP/S^+^Lep^−^ subgroup with a statistically significant difference (*P* = 0.002). As minor components, the lepidic patterns were a protective factor, and the solid and micropapillary components were poor factors. The recurrence was related to the presence of non-predominant patterns rather than their proportion. Adjuvant chemotherapy did not significantly improve the prognosis of the MP/S^+^Lep^-^ subgroup (*P* = 0.839).

**Conclusions:**

Regardless of the proportion, the presence of micropapillary/solid components and the absence of lepidic patterns are aggressive factors of DFS in patients with resected stage I acinar- or papillary-predominant lung adenocarcinoma.

## 1 Introduction

Non-small cell lung accounts for 85% of lung cancer and the most common histological type of which is lung adenocarcinoma. Histological classification of lung adenocarcinoma was established by the International Association for the Study of Lung Cancer, American Thoracic Society and European Respiratory Society (IASLC/ATS/ERS), which is an international standard for histologic subclassification of lung adenocarcinoma (1). Lung adenocarcinoma is comprised of five pathological subtypes including lepidic, acinar, papillary, micropapillary, and solid, of which the acinar-predominant component is the most common subtype accounting for about 40-50%. The lepidic pattern is categorized as low grade, acinar and papillary patterns as an intermediate grade, and micropapillary and solid patterns as high grade (2, 3). Many studies have reported a negative prognosis for solid- and micropapillary-predominant adenocarcinoma and a beneficial prognosis for patients with lepidic-predominant adenocarcinoma (4, 5). The DFS rate of lepidic predominant adenocarcinomas and the acinar/papillary predominant adenocarcinomas was 99% and 80.8-82.4% respectively, micropapillary and solid predominant lung adenocarcinomas have a poor prognosis with the 5-year DFS rate of approximately 33.3-73.6% (6).

Interestingly, recent studies have shown the minimal presence of lepidic components plays a protective role and micropapillary (7, 8) and solid components are risk factors in early-stage lung adenocarcinoma (9, 10), even if not as predominant patterns. Studies have shown that solid and micropapillary components account for 41.8% and 60.4% of early stage lung adenocarcinoma, and even in stage IA, solid and micropapillary components are still unfavorable factors even though they are not the predominant components (11–13). When the most predominant histologic pattern was intermediate-grade, the second most predominant pattern was high-grade, and recurrence risk increased by 4.2-fold compared with the low-grade group (14). However, in most cases, the composition of lung adenocarcinoma is complex and heterogeneous, with few lung adenocarcinomas having a pure component and most adenocarcinomas (80-90%) having a mixture of two or more growth patterns. In the real world, clinicians primarily consider the predominant pattern and pay little attention to the effect of minimal components in the prognosis of lung adenocarcinoma. Liu. et al. found that the recurrence hazard curve in early stage adenocarcinoma patients showed a typical “double-peaked” pattern. The first recurrence peak occurred 20–22 months after surgery and the second peak occurred 5–6 years after surgery (15). The DFS is more informative than over survival (OS) in predicting the biological behavior in early stage tumors. We explored the clinicopathological features of the non-predominant components and focused on the impact of lepidic, micropapillary, and solid as minimal components in the recurrence of intermediate-grade adenocarcinoma.

## 2 Patients and methods

### 2.1 Patients

We enrolled 153 patients with clinical stage T1-2aN0M0 stage I lung adenocarcinoma who underwent complete resection at Nanjing Chest Hospital from May 2014 to August 2017, and the follow-up deadline was August 2022. The inclusion criteria were as follows: 1. anatomical resection with standard mediastinal Lymph node dissection; 2. The pathological stages were determined according to the WHO eighth edition classification criteria; 3. The predominant subtype is acinar/papillary; 4. The minimal patterns include lepidic, solid, and micropapillary; Exclusion criteria were variants adenocarcinomas, adenocarcinomas *in situ* and minimally invasive adenocarcinomas, preoperative neoadjuvant chemotherapy or radiotherapy, positive surgical margin, a history of infection or other malignant tumors.

This study was approved by the Ethics Committee of Nanjing Brain Hospital. Informed consent was obtained from all participants in the study.

### 2.2 Histopathological evaluation

All resected specimens were formalin-fixed and stained with hematoxylin and eosin. The histologic classification of all slides was evaluated by two experienced pathologists according to IASLC/ATS/ERS classification criteria, respectively. The discrepancies were discussed to reach a consensus. Each histologic component present was recorded semiquantitatively in 5% increments. The non-predominant pattern was greater than or equal to 5% of the tumor. When the percentage of patterns was less than 5%, which was considered absent (16, 17). The predominant histological subtype was the highest proportion of the tumor, not necessarily 50% or greater (18, 19).

A total of 153 lung adenocarcinoma patients were classified into four groups based on the presence or absence of minimal patterns: MP/S^−^Lep^+^ group (micropapillary and solid components were absent, and lepidic components were present), MP/S^+^Lep^+^ group (either micropapillary or solid components were present, and lepidic components were present), MP/S^−^Lep^−^ group (both micropapillary, solid and lepidic components were absent) MP/S^+^Lep^−^ group (either micropapillary or solid components were present, and lepidic components were absent).

### 2.3 Methods

The histopathologic and clinicopathologic features of clinical data are as follows: gender, age, smoking history, surgical resection, predominant subtype, the proportion of micropapillary/solid components, the presence of lepidic and micropapillary/solid components, stage, differentiation, chemotherapy, laterality, visceral pleural invasion, lymphovascular invasion, DFS, OS, CEA, and PLR. The medical records and telephone were used for follow-up. A total of 153 patients were evaluated every 6 months for the first 2 years after surgery, and then once a year for the next 3-5 years. The last follow-up was in August 2022. The primary outcome was disease‐free survival (DFS). DFS was calculated from the date of surgery to the date of first recurrence or death from any cause, or last contact. The definition of overall survival (OS) was the time interval from surgery to the date of death or last contact. The normal value for CEA was < 5ug/l.

### 2.4 Statistical analysis

The categorical variables were summarized as frequencies and percentages, and the difference was analyzed by the chi-square test. The optimal cut-off value of PLR was determined by Youden’s Index and implemented through receiver operating characteristic (ROC). DFS was estimated by the Kaplan–Meier method and compared using the log-rank test. The Cox proportional hazards model was used to perform univariate and multivariate analyses. *P* < 0.05 values were considered statistically significant. The data were performed with SPSS 25.0 statistical software.

## 3 Results

### 3.1 The basic characteristics

The basic characteristics of the included patients were presented in **Table 1**. There were 55 females (35.9%) and 98 males (64.1%) with a median age of 60 years (31–84 years). Smokers were observed in 32 of 153 cases (20.9%). 146 patients (95.4%) underwent lobectomy and the remaining (n=7) underwent sublobar resection. Out of 153 patients, 88(57.5%) received postoperative adjuvant chemotherapy. Chemotherapy comprised platinum-based regimens, the platinum double‐drug chemotherapy programs were: pemetrexed in 73 cases (83.0%), docetaxel in 5 cases (5.7%), gemcitabine in 2 cases (2.3%), paclitaxel in 2 cases (2.3%), and unknown regimens in 6 cases (6.7%). The percentage of stage IA1, IA2, IA3 and IB patients was 3.9%, 44.4%, 30.1%, and 21.6%, respectively. 58 tumors (37.9%) were located in the left lung. The visceral pleural invasion was observed in 26 cases (17.0%) and lymphovascular invasion was seen in 60 cases (39.2%). Poor differentiation was observed in 74 cases (49.3%). CEA ≥5 ng/ml was found in 28 cases (19.0%).

**Table 1:** Clinicopathological characteristics of patients with stage I lung cancer.

Variable	N (%)
Gender	
Male	55 (35.9)
Female	98 (64.1)
Age at diagnosis(years)	
≥65	44 (28.8)
<65	109 (71.2)
Smoking history	
Smoker	32 (20.9)
Nonsmoker	121 (79.1)
Surgical resection	
Sublobar resection	7 (4.6)
Lobectomy	146 (95.4)
Predominant subtybe	
acinar	59 (38.6)
papillary	94 (61.4)
The proportion of MP/S+	
0%	55 (35.9)
5%-20%	50 (32.7)
20%-50%	48 (31.4)
Lepidic	
Absent	110 (71.9)
Present	43 (28.1)
Stage	
IA1	6 (3.9)
IA2	68 (44.4)
IA3	46 (30.1)
IB	33 (21.6)
Differentiation	
Well	76 (50.7)
Poorly	74 (49.3)
Chemotherapy	
Yes	88 (57.5)
No	65 (42.5)
Laterality	
Left	58 (37.9)
Right	95 (62.1)
Visceral pleural invasion	
Absent	127 (83.0)
Present	26 (17.0)
Lymphovascular invasion	
Absent	93 (60.0)
Present	60 (39.2)
Recurrence	
Yes	42 (27.5)
No	111 (72.5)
Survival	
Yes	7 (4.8)
No	140 (95.2)
CEA(ng/ml)	
<5.00	119 (81.0)
≥5.00	28 (19.0)
PLR	
<154.13	128 (84.2)
≥154.13	24 (15.8)

MP/S^+^, micropapillary/solid component were present; CEA, carcinoembryonic antigen; PLR, platelet-to-lymphocyte ratio.

For the predominant subtype, the frequency of papillary predominant adenocarcinoma was 61.4%, followed by acinar predominant adenocarcinoma (38.6%). As a non-predominant pattern, lepidic components were displayed in 43 cases (28.1%), solid and micropapillary patterns in 98 tumors (64.1%) according to the new IASLC histologic grading system. The proportion of high-grade patterns greater than 5% or equal to 5% and less than 20% was observed in 50 cases (32.7%). The proportion of high-grade patterns greater than 20% or equal to 20% and less than 50% was observed in 48 cases (31.4%). The percentage of MP/S^−^Lep^+^, MP/S^+^Lep^+^, MP/S^−^Lep^−^ and MP/S^+^Lep^−^ group was 19.0%, 9.2%, 17.0%, and 54.9%, respectively. The median time to follow up was 55.3 months (interquartile range, 56.5 to 67 months). In the follow-up period, 27.5% of patients experienced recurrence and 4.8% of patients occurred death.

### 3.2 Optimal cut-off value for ROC curve

**Figure 1:**
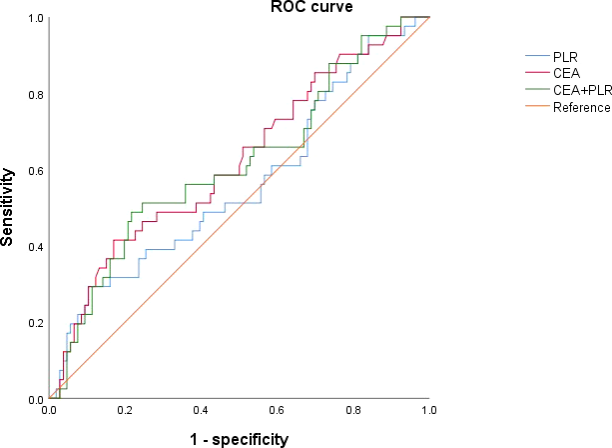
Receiver operating characteristic curves for analysis of using CEA and PLR in predicting disease-free survival.

A cut-off value of 154.1 was used to discriminate between patients with high and low preoperative PLR, with AUC of 0.561 (sensitivity: 0.286, specificity: 0.892). Patients were distributed into PLR high groups (n = 24) and low PLR groups (n =128). PLR and CEA were combined for diagnosis, and the cut off value was 0.298 (sensitivity: 0.488; specificity: 0.783; AUC: 0.612), which can slightly enhance the diagnostic performance (**Figure 1**).

### 3.3 Relationships between clinicopathological characteristics and the risk subgroups based on non-predominant components

**Figure 2:**
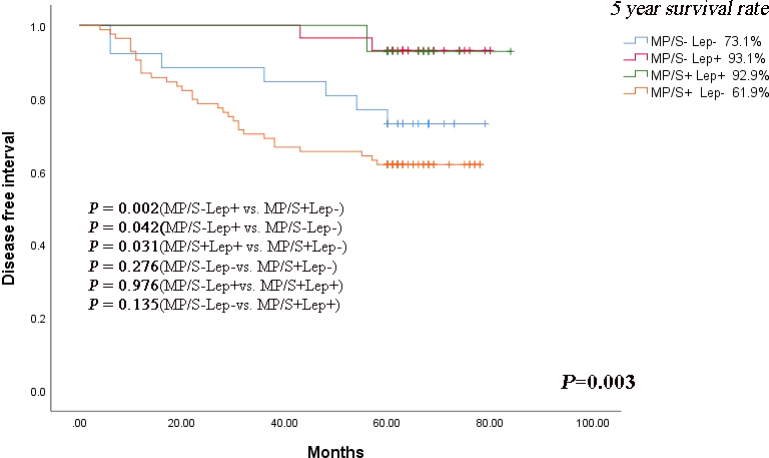
Kaplan-Meier curve of DFS according to lepidic, micropapillary/solid minor components in stage I lung adenocarcinoma patients. The comparison of DFS between 2 groups: MP/S^−^Lep^+^ vs. MP/S^+^Lep^−^ group (*P* = 0.002), MP/S^−^Lep^+^ vs. MP/S^−^Lep^−^ group (*P*=0.042), MP/S^+^Lep^+^ vs. MP/S^+^Lep^−^ group (*P* = 0.031), MP/S^−^Lep^−^ vs. MP/S^+^Lep^−^ group (*P* = 0.276), MP/S^−^Lep^+^ vs. MP/S^+^Lep^+^ group (*P* = 0.976), MP/S^-^Lep^-^ vs. MP/S^+^Lep^+^ group (*P* = 0.135).

The relationship between the risk subgroup divided according to the minor components and clinicopathological features was shown in **Table 2**. The risk groups based on non-predominant components were associated with differentiation (*P* = 0.001). The MP/S^+^Lep^-^ group was vulnerable to poorly differentiation. The minor pattern was related to Lymphovascular invasion (*P* = 0.001). The MP/S^+^Lep^-^ group was more susceptible to Lymphovascular invasion. The minor pattern was significantly correlated with postoperative recurrence (*P* = 0.003). The 5-year DFS rates of MP/S^−^Lep^+^, MP/S^+^Lep^+^, MP/S^−^Lep^−^ and MP/S^+^Lep^−^ group were 93.1%, 92.9%, 73.1%, 61.9%, respectively. The survival time of MP/S^−^Lep^+^ group was longer than MP/S^+^Lep^−^ group with a statistically significant difference (*P* = 0.002) (**Figure 2**). It indicated the presence of micropapillary/solid components and absence of lepidic patterns are poor prognostic factors of DFS in patients with resected stage I acinar- or papillary-predominant lung adenocarcinoma.

**Table 2:** Relationships between clinicopathological characteristics and the presence of lepidic and micropapillary/solid pathological patterns as minor components in patients with stage I lung adenocarcinoma.

				Risk Group			
Variables	N	MP/S^−^Lep^+^ (%)	MP/S^+^Lep^+^(%)	MP/S^−^Lep^−^ (%)	MP/S^+^Lep^-^(%)	χ^2^	*P* v a l u e
Gender						2.994	0.393
Female	98	21 (21.4)	9 (9.2)	19 (19.4)	49 (50.0)		
Male	55	8 (14.5)	5 (9.1)	7 (12.7)	35 (63.3)		
Age at diagnosis (years)						4.813	0.186
≥65	44	8 (18.2)	6 (13.6)	10 (22.7)	20 (45.5)		
<65	107	21 (19.6)	6 (5.6)	16 (15.0)	64 (59.8)		
Smoking history						0.326	0.955
Smoker	32	5 (15.6)	3 (9.4)	6 (18.8)	18 (56.3)		
Nonsmoker	121	24 (19.8)	11 (9.1)	20 (16.5)	66 (54.5)		
Predominant subtype						7.002	0.072
acinar	59	15 (25.4)	7 (11.9)	5 (8.5)	32 (54.2)		
papillary	94	14 (14.9)	7 (7.4)	21 (22.3)	52 (55.3)		
Stage						7.406	0.060
IA	120	25 (20.8)	14 (11.7)	21 (17.5)	60 (50.0)		
IB	33	4 (12.1)	0 (0.0)	5 (15.2)	24 (72.7)		
Differentiation						38.241	**0.001**
Poorly	74	4 (5.4)	6(8.1)	5 (6.8)	59 (79.7)		
Well	76	23 (30.3)	8(10.5)	21 (27.6)	24 (31.6)		
Chemotherapy						3.953	0.267
Yes	88	13 (14.8)	7 (8.0)	14 (15.9)	54 (61.4)		
No	65	16 (24.6)	7 (10.8)	12 (18.5)	30 (46.2)		
Laterality						5.845	0.119
left	58	13 (22.4)	5 (8.6)	14 (24.1)	26 (44.8)		
Right	135	16 (11.9)	9 (6.7)	26 (19.3)	84 (62.2)		
Visceral pleural invasion						0.408	0.939
Absent	127	23 (18.1)	12 (9.4)	22 (17.3)	70 (55.1)		
Present	26	6 (23.1)	2 (7.7)	4 (15.4)	14 (53.8)		
Lymphovascular invasion						26.284	**0.001**
Absent	93	26 (28.0)	8 (8.6)	22 (23.7)	37 (39.8)		
Present	60	3 (5.0)	6 (10.0)	4 (6.7)	47 (78.3)		
Recurrence						13.834	**0.003**
Yes	42	2 (4.8)	1 (2.4)	7 (16.7)	32 (76.2)		
No	111	27 (24.3)	13 (11.7)	19 (17.1)	52 (46.8)		
Survival						3.020	0.389
Yes	7	0(0.0)	0 (0.0)	2 (28.6)	5 (71.4)		
No	140	28 (20.0)	14 (10.0)	24 (17.1)	74 (52.9)		
CEA						5.078	0.166
Low	119	25 (21.0)	11 (9.2)	21 (17.6)	62 (52.1)		
High	28	3 (10.7)	2 (7.1)	2 (7.1)	21 (75.0)		
PLR						4.960	0.175
Low	128	23 (18.0)	14 (10.9)	23 (18.0)	68 (53.1)		
High	24	6 (25.0)	0 (0.0)	2 (8.3)	16 (66.7)		

CEA, carcinoembryonic antigen; PLR, platelet-to-lymphocyte ratio; MP/S^−^Lep^+^ (micropapillary/solid components were absent and lepidic components were present); MP/S^+^Lep^+^ (both micropapillary/solid component and lepidic component were present); MP/S^−^Lep^−^ (both micropapillary/solid component and lepidic component were absent); MP/S^+^Lep^−^ (micropapillary/solid component were present and lepidic component were absent). The bold values statistical significance at P < 0.05 level.

In the patients without micropapillary/solid patterns, the lepidic components predicted a better prognosis compared with the non-lepidic components (*P*=0.042). The same result was seen in the patients with micropapillary/solid patterns (*P* = 0.031). The results suggested that lepidic components played a protective role in the early stage acinar- or papillary-predominant lung adenocarcinoma. In the patients with or without the lepidic components, the solid and micropapillary components showed no beneficial effect on prognosis (*P* = 0.976 and *P* = 0.276, respectively).

Nevertheless, we found the MP/S^-^Lep^-^ group had shorter DFS than MP/S^+^Lep^+^ group, but there was no statistically significant difference in the 5-year DFS rate between the two groups (*P* = 0.135), indicating there was no statistically significant difference in the rate of relapse when the high grade and low grade were both present or absent together. The minor components were related to aggressive factors such as poorly differentiation and lymphovascular invasion. The MP/S^+^Lep^-^ group was more vulnerable to recurrence compared with other subgroups.

### 3.4 The prognostic factors of disease-free survival in univariate analysis and multivariate analysis

Univariate analysis indicated that non-predominant patterns (*P* = 0.014), lymphovascular invasion (*P* = 0.001), elevated CEA level (*P* = 0.001), and elevated PLR level (*P* = 0.012) were the potential predictive factors for the stage I acinar- or papillary- predominant adenocarcinoma.

Multivariate Cox regression analysis further revealed that MP/S^+^Lep^-^ group (*P* = 0.043) and elevated PLR level (*P* = 0.032) were independent risk factors of DFS (**Table 3**).

**Table 3:** Univariate and multivariate COX regression analysis for disease-free survival in stage I lung adenocarcinoma.

		Disease Free	Survival	
	Univariate		Multivariate	
**Variables**	HR (95%CI)	***P-*value**	HR (95%CI)	***P-*value**
**Age**(≥65 vs.<65)	0.906 (0.456-1.804)	0.780		
**Gender** (male vs. female)	0.857 (0.460-1.598)	0.627		
**Smoking history** (cur/for vs. never)	1.550 (0.779-3.084)	0.212		
**Predominant subtybe** (acinar vs. papillary)	0.720 (0.392-1.321)	0.289		
Group				
MP/S^+^Lep^-^	Ref			
MP/S^-^Lep^-^	0.634 (0.280-1.437)	0.275	0.949 (0.375-2.400)	0.912
MP/S^-^Lep^+^	0.144 (0.034-0.599)	**0.008**	0.220 (0.051-0.955)	**0.043**
MP/S^+^Lep^+^	0.148 (0.020-1.087)	0.060	0.242 (0.032-1.821)	0.168
**Stage** (IB vs. IA)	3.262 (1.759-6.051)	**0.001**	1.564 (0.755-3.239)	0.229
**Differentiation** (Poorly vs. Well)	1.213 (0.657-2.242)	0.537		
**Lymphovascular invasion** (present vs. absent)	2.806 (1.513-5.203)	**0.001**	1.761 (0.883-3.514)	0.108
**Visceralpleural invasion** (absent vs. present)	0.802 (0.371-1.733)	0.575		
**Surgical resection** (Sublobar resection vs. Lobectomy)	0.462 (0.064-3.359)	0.446		
**Laterality** (Left vs. right)	0.590 (0.302-1.152)	0.122		
**Chemotherapy** (yes vs. no)	1.292 (0.693-2.408)	0.421		
**CEA**(high vs. low)	2.922 (1.529-5.586)	**0.001**	2.094 (0.983-4.464)	0.056
**PLR** (high vs. low)	2.365 (1.209-4.628)	**0.012**	2.179 (1.068-4.445)	**0.032**

CEA, carcinoembryonic antigen; PLR, platelet-to-lymphocyte ratio; MP/S^−^Lep^+^ (micropapillary/solid components were absent and lepidic components were present); MP/S^+^Lep^+^ (both micropapillary/solid component and lepidic component were present); MP/S^−^Lep^−^ (both micropapillary/solid component and lepidic component were absent); MP/S^+^Lep^−^ (micropapillary/solid component were present and lepidic component were absent). The bold values statistical significance at P < 0.05 level.

### 3.5 Subgroup analysis of minimal components

**Figure 3:**
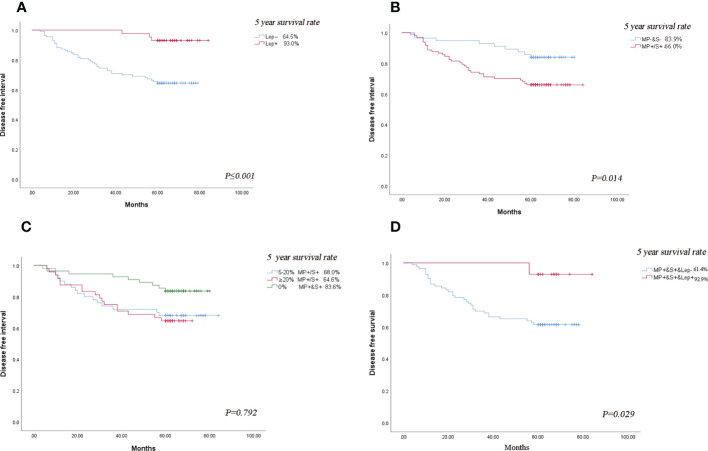
Subgroup analysis. **(A)** Comparison of DFS in patients with lung adenocarcinoma based on presence or absence of lepidic minor component. **(B)** Comparison of DFS in patients with lung adenocarcinoma based on the presence or absence of micropapillary/solid minor components component. **(C)** Comparison of DFS in patients with lung adenocarcinoma based on the proportion of micropapillary/solid minor components component. **(D)** Comparison of the effect of the presence or absence of lepidic component on DFS in patients with the micropapillary/solid minor component. The symbol of “&” means and.

We categorized acinar/papillary-predominant lung adenocarcinoma according to the presence of lepidic patterns. There were 28.1% (n = 43) patients in the group with the presence of lepidic patterns, the remaining was 71.9% (n =110) patients. The 5-year DFS rates of the group with the presence of lepidic patterns were 93.0% and the group with the absence of lepidic patterns was 64.5%. Therefore the lepidic pattern was a positive factor in stage I lung adenocarcinoma (**Figure 3A**). The non-predominant components were divided into two groups according to the presence of solid and micropapillary components: MP^+^/S^+^ subtype (either micropapillary or solid component was present) and MP^-^&S^-^ subtype (both micropapillary and solid components were absent). The MP^+^/S^+^ subtype was present in 63.3% (n = 97) of patients and MP^-^&S^-^ subtype was observed in 36.7% (n = 56) of patients. The 5-year DFS rates of MP^+^/S^+^ subtype and MP^-^&S^-^ subtype were 66.0% and 83.9%, respectively, and the difference was significant (*P* = 0.014). The solid and micropapillary patterns were associated with poor outcomes (**Figure 3B** **)**.

The 2020 IASLC/ATS/ERS grading system proposed a cut-off of 20% for high-grade patterns as a risk factor (2). According to the proposal of the new IASA system, we classified the total proportion of solid and micropapillary components (TPSM) in tumors into two groups in our study: patients with 5-20% proportion of solid and micropapillary were defined as TPSM-low (n=50) and patients with 20%-50% proportion of solid and micropapillary were defined as TPSM-high (n = 48). The 5-year DFS rates in TPSM-low and TPSM-high were 68.0% and 64.6%, respectively. The result showed there was no significant difference between the two groups (*P* = 0.792) (**Figure 3C**). The presence of solid/micropapillary patterns rather than the proportion of solid/micropapillary patterns affected the DFS in lung adenocarcinoma.

For Lung adenocarcinoma with MP^+^/S^+^ subtype, the DFS rates in patients with the presence of lepidic patterns and absence of those were 92.9% and 61.4%, respectively. The results demonstrated that even in patients with poor prognosis, the presence of lepidic patterns could improve survival significantly (*P* = 0.029) (**Figure 3D**). The published literature only took the solid and micropapillary components into account, or just considered the lepidic component alone. In the future, we should emphasize the inclusion of all three components simultaneously when assessing the prognosis of lung adenocarcinoma.

**Figure 4:**
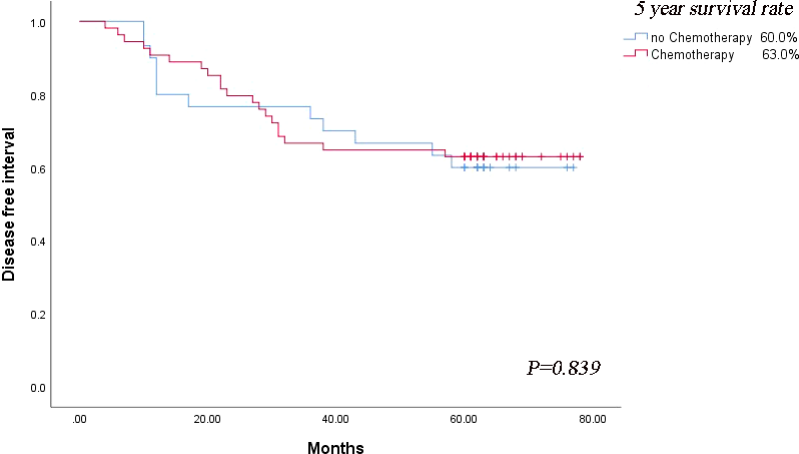
Survival outcomes regarding disease-free survival based on the strategy of adjuvant chemotherapy.

The MPS^-^/Lep^+^ group had the adverse outcome among those with acinar- or papillary-predominant lung adenocarcinoma, hence we explored whether these patients could benefit from adjuvant chemotherapy. In terms of the prognosis of patients in MPS^-^/Lep^+^ group, the DFS rate was no significant difference between postoperative chemotherapy and non-postoperative chemotherapy (*P* = 0.839) (**Figure 4**).

## 4 Discussion

The MP/S^+^Lep^-^ and high PLR level were determined as poor risks in acinar/papillary-predominant lung adenocarcinoma patients with tumor size ≤4 cm. The MP/S^+^Lep^-^ group was prone to recurrence, which may be strongly associated with poorly differentiation and lymphovascular invasion. Adjuvant chemotherapy did not significantly improve DFS in the MP/S^+^Lep^-^ subgroup. The above results reflected that even for the same predominant histopathological subtype, the prognosis varied with the diverse composition of the minimal patterns. It is advised to concentrate on all growth patterns observed in tumors beyond predominant components. Zhao et al. also concluded not only predominant subtypes but also minor components had an important value in clinical outcomes (20). Mäkinen et al. reported that the non-predominant lepidic components were related to a favorable outcome in invasive adenocarcinoma (21). A study indicated solid minor components and solid predominant subtypes both predicted a worse prognosis compared with the absent solid pattern. Chemotherapy was beneficial for solid predominant components rather than solid as minor patterns (22). The MP/S^+^Lep^-^ was an independent factor for DFS and OS, and stage IA patients in the MP/S^+^Lep^-^ subgroup did not benefit from chemotherapy. Those findings are supported by our results (23).

We found that patients in the MP/S^+^Lep^-^ subgroup were closely connected with poorly differentiation and lymphovascular invasion. Perhaps this can be explained by the following theoretical mechanism. The solid/micropapillary patterns include increased laminin-5 expression levels, which is an extracellular matrix protein that is crucial in cell migration, intercellular adhesion, and tumor invasion reflecting the biologically aggressive nature of tumors (24). The presence of lepidic patterns indicated lower cancer cell-specific expression levels of hypoxia markers and a smaller number of tumor-promoting stromal cells (25, 26). The presence of solid and micropapillary patterns were related to metastatic lymph nodes, inversely, the presence of lepidic patterns predicted nonmetastatic lymph nodes (27). In conclusion, the lung adenocarcinoma with the presence of solid/micropapillary components and the absence of lepidic pattern presented the worse DFS. To the best of our knowledge, this is the first study investigating the relationship between PLR and non-predominant components on survival.

We noticed that the presence of non-predominant patterns rather than their proportion was associated with recurrence in stage I acinar/papillary-predominant lung adenocarcinomas. Chen et al. reported the proportion of solid or micropapillary patterns was not related to recurrence in IA lung adenocarcinoma (28). As a result, identifying the presence of a solid or micropapillary component is more valuable than determining the percentage of either of these components. A prognostic nomogram according to a new classification of combined micropapillary and solid components revealed that patients with a total proportion of solid and micropapillary components ≥40% in stage IA patients had shorter DFS and OS compared with less than the total proportion (29). Our conclusions contradict the above findings, maybe due to our small sample size.

In our study, the MP/S^+^Lep^-^ group could not benefit from adjuvant chemotherapy. Cao et al. also reported that adjuvant chemotherapy is beneficial for solid predominant patterns of stage IB lung adenocarcinoma, while those with solid minor patterns will not (30). The low relapse rates in the non‐predominant population especially of stage I tumors and limited sample size may explain the failure of adjuvant chemotherapy as a prognostic factor.

There are some limitations in this study. Firstly, owing to the short follow-up period plus the small number of participants, it was difficult to perform the analysis of OS, and we are working to enlarge the sample size. Secondly, the data collection of this study is retrospective, there is an imbalance in the distribution of histological grades, so it is urgent to conduct multi-institutional randomized clinical trials to verify the results. Additionally, we did not evaluate the status of EGFR and KRAS mutation and the PD-L1 expression to explore whether the immunotherapy or targeted therapy could benefit the MP/S^+^Lep^-^ group. The results show the emphasis should be placed on the non-predominant histological subtype and the necessity of classifying acinar/papillary-predominant adenocarcinomas into different risk groups so as to select high-risk subgroups of patients to administer more intensive surveillance.

## Data availability statement

The raw data supporting the conclusions of this article will be made available by the authors, without undue reservation.

## Ethics statement

This study was approved by the Ethics Committee of Nanjing Brain Hospital. Informed consent was obtained from all participants in the study. The patients/participants provided their written informed consent to participate in this study. Written informed consent was obtained from the individual(s) for the publication of any potentially identifiable images or data included in this article.

## Author contributions

WL and QZ searched the database, judged study eligibility, and extracted data. WL and TZ analyzed data and wrote the paper. CX and LLdesigned the study and revised this paper. All authors contributed to the article and approved the submitted version.
